# *PDCD1* (*PD-1*) promoter methylation predicts outcome in head and neck squamous cell carcinoma patients

**DOI:** 10.18632/oncotarget.17354

**Published:** 2017-04-21

**Authors:** Diane Goltz, Heidrun Gevensleben, Joern Dietrich, Friederike Schroeck, Luka de Vos, Freya Droege, Glen Kristiansen, Andreas Schroeck, Jennifer Landsberg, Friedrich Bootz, Dimo Dietrich

**Affiliations:** ^1^ Institute of Pathology, University Hospital Cologne, Cologne, Germany; ^2^ Institute of Pathology, University Hospital Bonn, Bonn, Germany; ^3^ Department of Otolaryngology, Head and Neck Surgery, University Hospital Bonn, Bonn, Germany; ^4^ Ear, Nose and Throat Clinic, University Hospital Essen, Essen, Germany; ^5^ Department of Dermatology and Allergy, University of Bonn, Bonn, Germany

**Keywords:** PD-1, PDCD1, HPV, head and neck squamous cell carcinoma, DNA methylation

## Abstract

**Background:**

Biomarkers that facilitate the prediction of disease recurrence in head and neck squamous cell carcinoma (HNSCC) may enable physicians to personalize treatment. In the current study, DNA promoter methylation of programmed cell death 1 (*PDCD1, PD-1*) was evaluated as a prognostic biomarker in HNSCC patients.

**Results:**

High *PDCD1* methylation (m*PDCD1*) was associated with a significantly shorter overall survival after surgical resection in both the discovery (HR = 2.24 [95%CI: 1.08–4.64], *p* = 0.029) and the validation cohort (HR = 1.54 [95%CI: 1.08–2.21], *p* = 0.017). In multivariate Cox proportional hazards analysis, *PDCD1* methylation remained a significant prognostic factor for HNSCC (HR = 2.14 [95%CI: 1.19–3.84], *p* = 0.011). Further, m*PDCD1* was strongly associated with the human papilloma virus (HPV) status.

**Materials and Methods:**

m*PDCD1* was assessed retrospectively in a discovery cohort of 120 HNSCC patients treated at the University Hospital of Bonn and a validation cohort of 527 HNSCC cases analyzed by The Cancer Genome Atlas Research Network.

**Conclusions:**

**PDCD1**

methylation might aid the identification of HNSCC patients potentially benefitting from a radical or alternative treatment, particularly in the context of immunotherapies targeting PD-1/PD-L1.

## INTRODUCTION

Head and neck squamous cell carcinoma (HNSCC) is a major cause of death in Western countries accounting for an estimated incidence of 62,000 and 13,000 related deaths in the US in 2016 [1]. Major risk factors comprise exposure to chemical carcinogens such as tobacco and alcohol [2]. In addition, high-risk types of the human papilloma virus (HPV) are estimated to cause one fourth of head and neck cancer cases [3–7]. In over 90% of HPV-associated HNSCCs, HPV type 16 is identified as the causative agent [8]. Several studies have demonstrated that HPV-positive (HPV^+^) and HPV-negative (HPV^−^) HNSCCs are separate entities associated with distinct etiology, clinical behaviour, treatment outcomes, imaging, pathological appearance, and molecular profiles [5, 9, 10].

Despite intensive local treatment, HNSCCs generally have an unsatisfactory prognosis due to the high percentage of locoregional tumor recurrence and distant metastasis [11]. As a consequence, these tumors do not only require the standard surgical and radiation treatments but additional effective systemic treatment. During the last couple of years, immunomodulatory therapies have increasingly emerged as a promising new treatment option for advanced malignancies. New insights on the interaction between tumor and host immune response have been particularly focusing on the programmed death-1 receptor (PD-1)/programmed death-1 ligand (PD-L1) pathway as potential therapy target in various tumor entities. Although HNSCC has traditionally been considered to be a very immunosuppressive or at least non-immunogenic tumor type, recent results from clinical studies of immune checkpoint modulating drugs have led to a resurgence of enthusiasm for immunotherapeutic approaches [12]. Currently, a variety of clinical trials and substances for the treatment of HNSCC are underway, primarily focussing on targeting and inhibiting the PD-1/PD-L1 axis [10]. Recently, the PD-1 checkpoint inhibitor pembrolizumab has gained regulatory approval for the treatment of recurrent/metastasized HNSCC [12]. So far, cancer immunotherapy with immune checkpoint modulating drugs seems to be independent of HPV status and may be successful even in PD-L1 low level expressing tumors [12, 13]. Robust predictive markers for patient selection, however, are not yet available [13].

Of note, Lyford-Pike *et al*. demonstrated that the PD-1 receptor ligand PD-L1 is differentially expressed among HPV^+^ and HPV^-^ HNSCC [14], corroborating the role of the PD-1/PD-L1 pathway in HPV-related HNSCC immune resistance. While PD-1 receptor ligand PD-L1 has been shown to be expressed in various types of cancers [15], the immune inhibitory receptor PD-1 (also known as CD279 or PDCD1), a member of the extended CD28/CTLA-4 family, is known to be stably expressed only on T cells exposed to a chronic antigen [16, 17]. PD-1 expression has further been shown to be regulated by promoter methylation [18] and to be associated with biochemical recurrence-free survival in prostate cancer patients. Encouraged by these recent findings, we aimed to further complete the insight of the PD-1/PD-L1 pathway's activity in HNSCC.

## RESULTS

### *PDCD1* promoter methylation in HNSCC patients (discovery cohort)

For the analysis of *PDCD1* methylation (m*PDCD1*) in the discovery cohort, a quantitative methylation-specific qPCR targeting the *PDCD1* promoter region (Supplementary Figure 1) was used as recently described [19]. Median m*PDCD1* of all HNSCC tumor tissues was 28.6%. The distribution of m*PDCD1* levels did not differ across all categories of tumor location (mouth, oropharynx, hypopharynx, and larynx, Table 1A). Women presented with significantly higher m*PDCD1* levels compared to men. Analysis of prognostic clinicopathological variables showed no correlation with age at initial diagnosis, tumor grade, pathologic T (pT) and N (pN) categories, or metastasis (Table 2). P16 expression as a surrogate marker for HPV-related HNSCC, however, showed a strong negative association with m*PDCD1*. Inversely, a history of smoking significantly correlated with m*PDCD1* (Table 2).

**Table 1A:** Association of PDCD1 promoter methylation with clinicopathological data: Discovery cohort

Variable	All Patients	[%]	Median m*PDCD1*	*p*-value	m*PDCD1_low_*	[%]	m*PDCD1_high_*	[%]	*p*-value
**Patient number**	120	100	28.6		40	33.3	80	66.7	
**Sex**									
Female	26	21.7	42.2		5	19.2	21	80.8	
Male	94	78.2	27.4	0.019*	35	37.2	59	62.8	0.085^Χ^
**Patients with follow-up**	115	95.8							
Mean [Months]	32 [29–34]								
Median [Months]	25								
Range [Months]	0-115								
**Localization**									
Oral cavity	23	19.2	29.0		6	26.1	17	73.9	
Oropharynx	53	44.2	22.8		25	47.2	28	52.8	
Hypopharynx	9	7.5	27.3		3	33.3	6	66.7	
Larynx	35	29.2	32.4	0.15^ε^	6	17.1	29	82.9	0.026^Χ^
Lip	0	0							
**Age [years]**									
Mean	61.9 [59.1–62.8]								
Median	61								
n < Median	61	50.8	27.8		22	36.1	39	63.9	
n > Median	59	49.2	29.7	0.24*	18	30.5	41	69.5	0.52^Χ^
Unknown									
**pT-category**									
pTis.pT1/2	56	46.7	26.6		19	33.9	37	66.1	
pT3/4	43	35.8	28.8	0.50*	15	34.9	28	65.1	0.92^Χ^
Unknown	21	17.5							
**Nodal status**									
pN0	43	35.8	29.7		11	25.6	32	74.4	
pN1	17	14.2	29.5		4	23.5	13	76.5	
≥ pN2	60	50	26.2	0.36^ε^	25	41.7	35	58.3	0.15^Χ^
Unknown	0								
**Tumor grade**									
G1	2	1.7	35.6		1	50.0	1	50.0	
G2	64	53.3	28.9		19	29.7	45	70.3	
G3	29	79.2	36.0	0.75^ε^	9	31.0	20	69.0	0.83^Χ^
G4	0	0							
Unknown	25	20.8							
**Distant metastasis**									
cM0	117	97.5	28.8		40	34.2	77	65.8	
cM1	2	1.7	33.1	0.85*	0	0	2	100	0.55^ζ^
Unknown	1	0.8							
**Surgical margin**									
R0	86	71.7	28.4		28	32.6	58	67.4	
R1	13	10.8	38.9	0.51*	4	30.8	9	69.2	0.90^Χ^
Unknown	21	17.5							
**HPV (p16)**									
p16-negative	64	53.3	37.3		15	23.4	49	76.6	
p16-positive	15	12.5	15.9	0.002*	10	66.7	5	33.3	0.001^Χ^
Unknown	41	34.2							
**Smoking history**									
Negative	13	10.8	15.9		8	61.5	5	38.5	
Positive	82	68.3	29.0	0.044*	27	32.9	55	67.1	0.047^Χ^
Unknown	25	20.8			8.5				

* - Mann-Whitney-Test; e - Kruskal Wallis; c - χ^2^ –test; ζ – Fisher's exact test

**Table 1B:** Association of *PDCD1* promoter methylation with clinicopathological data: Validation cohort

Variable	All Patients	[%]	Median m*PDCD1*	*p-*value	m*PDCD1*_low_	[%]	m*PDCD1_high_*	[%]	*p-*value	Missing immune cell content	[%]	Median B cell	*p-*value	Median CD4pos T cells	*p-*value	Median CD8pos T cells	*p-*value
**Patient number**	527	100	26.6		174	33	353	67		13	2.5	0.06		0.11			
**Sex**																	
Female	143	27.1	27.8		40	28	103	72		6	4.2	0.062		0.108		0.15	
Male	384	72.9	26.9	0.13*	134	34.9	250	65.1	0.13^Χ^	7	1.8	0.059	0.82*	0.11	0.77*	0.142	0.27*
**Localization**																	
Oral cavity	345	65.5	26.7		109	31.6	236	68.4		8	2.3	0.052		0.106		0.133	
Oropharynx	55	10.4	19.8		37	67.3	18	32.7		4	7.3	0.156		0.142		0.25	
Hypopharynx	10	1.9	34.7		3	30	7	70		1	10	0.055		0.132		0.122	
Larynx	114	221.6	28.3		23	20.2	91	79.8		0	0	0.071		0.111		0.147	
Lip	3	0.6	13.4	<0.001^ε^	2	66.7	1	33.3	<0.001^Χ^	0	0	0.12	<0.001^ε^	0.122	0.013ε	0.191	0.001ε
**Age [years]**																	
Mean	60.9 [59.9-61.9]																
Median	61																
n < Median	281	53.3	25.8		109	38.8	172	61.2		6	2.1	0.061		0.11		0.139	
n > Median	245	46.5	27.8	0.002*	65	26.5	180	73.5	0.003^Χ^	7	2.9	0.056	0.24*	0.108	0.51*	0.148	0.48*
Unknown	1	0.2															
**pT category**																	
pTis.pT1/2	191	36.2	25.7		71	37.2	120	62.8		5	2.6	0.065		0.117		0.147	
pT3/4	274	52	27.8	0.014*	73	26.6	201	73.4	0.92^Χ^	6	2.2	0.052	0.005*	0.1	0.001*	0.14	0.18*
Unknown	62	11.8															
**pN category**																	
pN0	179	34	27.6		51	28.9	128	71.5		5	2.8	0.057		0.103		0.139	
pN1	68	12.9	25.7		18	26.5	50	73.5		2	2.9	0.063		0.121		0.149	
≥pN2	172	32.6	26.6	0.86^ε^	59	34.3	113	65.7	0.15^Χ^	4	2.3	0.059	0.71^ε^	0.109	0.34ε	0.14	0.77ε
Unknown	108	20.5															
**Tumor grade**																	
G1	64	12.1	28.3		16	25	48	75		2	3.1	0.057		0.1		0.133	
G2	312	59.2	27.3		86	27.6	226	72.4		10	3.2	0.055		0.101		0.139	
G3	122	23.1	26.5		56	44.8	69	55.2		0	0	0.072		0.129		0.162	
G4	7	1.3	18.7	0.029^ε^	6	85.7	1	14.3	<0.001^Χ^	0	0	0.251	0.001^ε^	0.291	<0.001ε	0.311	0.035ε
Unknown	22	4.2															
**Distant metastasis**																	
cM0	495	93.9	26.5		164	33.1	331	66.9		12	2.4	0.061		0.111		0.145	
cM1	6	1.1	36.1	0.081*	1	16.7	5	83.3	0.68^Χ^	0	0	0.035	0.48*	0.966	0.38*	0.127	0.20*
Unknown	26	4.9															
**Surgical margin**																	
R0	404	76.7	26.9		124	30.7	280	69.3		10	2.5	0.059		0.11		0.141	
R1	62	11.8	25.8	0.50*	22	35.5	40	64.5	0.45^Χ^	1	1.6	0.043	0.43*	0.95	0.52*	0.139	0.94*
Unknown	61	11.6															
**HPV**																	
HPV-	74	14	28		19	25.7	55	74.3		2	2.7	0.053		0.114		0.145	
HPV+	42	8	19.6	<0.001*	29	69	13	31	<0.001^Χ^	5	11.9	0.155	<0.001*	0.146	0.12*	0.265	0.003*
Unknown	411	78															
**Smoking history**																	
vNegative	122	23	25.5		49	40.2	73	59.8		7	5.7	0.061		0.105		0.15	
Positive	392	74.4	26.8	0.13*	122	31.1	270	68.9	0.064^Χ^	5	1.3	0.06	0.83*	0.11	0.80*	0.143	0.58*
Unknown	13	2.5															

* - Mann-Whitney U test; ε - Kruskal-Wallis test; χ - χ^2^ –Test

**Table 2:** Correlation of PDCD1 promoter methylation with clinicopathological parameters

	Discovery cohort		Validation cohort	
Variable	Spearman's ρ	*p*-value	Spearman's ρ	*p*-value
Age	0.053	0.56	0.110	0.012^¥^
Pathological T category (pT)	0.055	0.59	0.148	0.001^¥^
Pathological nodal status (pN)	–0.118	0.12	–0.026	0.60
Distant metastasis (cM)	0.019	0.84	0.078	0.081
Tumor grade (WHO 2006)	0.070	0.50	–0.107	0.016^¥^
HPV association	–0.352	0.001^¥^	–0.404	< 0.001^¥^
History of smoking	0.208	0.043^¥^	0.068	0.13
B lymphocytes	NA	NA	–0.175	< 0.001^¥^
CD4*^pos^* T lymphocytes	NA	NA	–0.118	0.007^¥^
CD8*^pos^* T lymphocytes	NA	NA	–0.095	0.031^¥^
Macrophages	NA	NA	0.005	0.90
Dendritic cells	NA	NA	–0.073	0.10

*P*-values and correlation coefficients (Spearman's r) are shown. *PDCD1* methylation is analyzed as a continuous variable

^¥^ significant feature; NA: not available.

Since differential m*PDCD1* was related to PD-1 expression in lymphocytes [18] and epithelial tumors are generally devoid of PD-1 expression [19], we speculated that differential m*PDCD1* might reflect PD-1 regulation in the immune compartment. However, the number of cases did not guarantee sufficient power to support this assumption.

**Figure 1:**
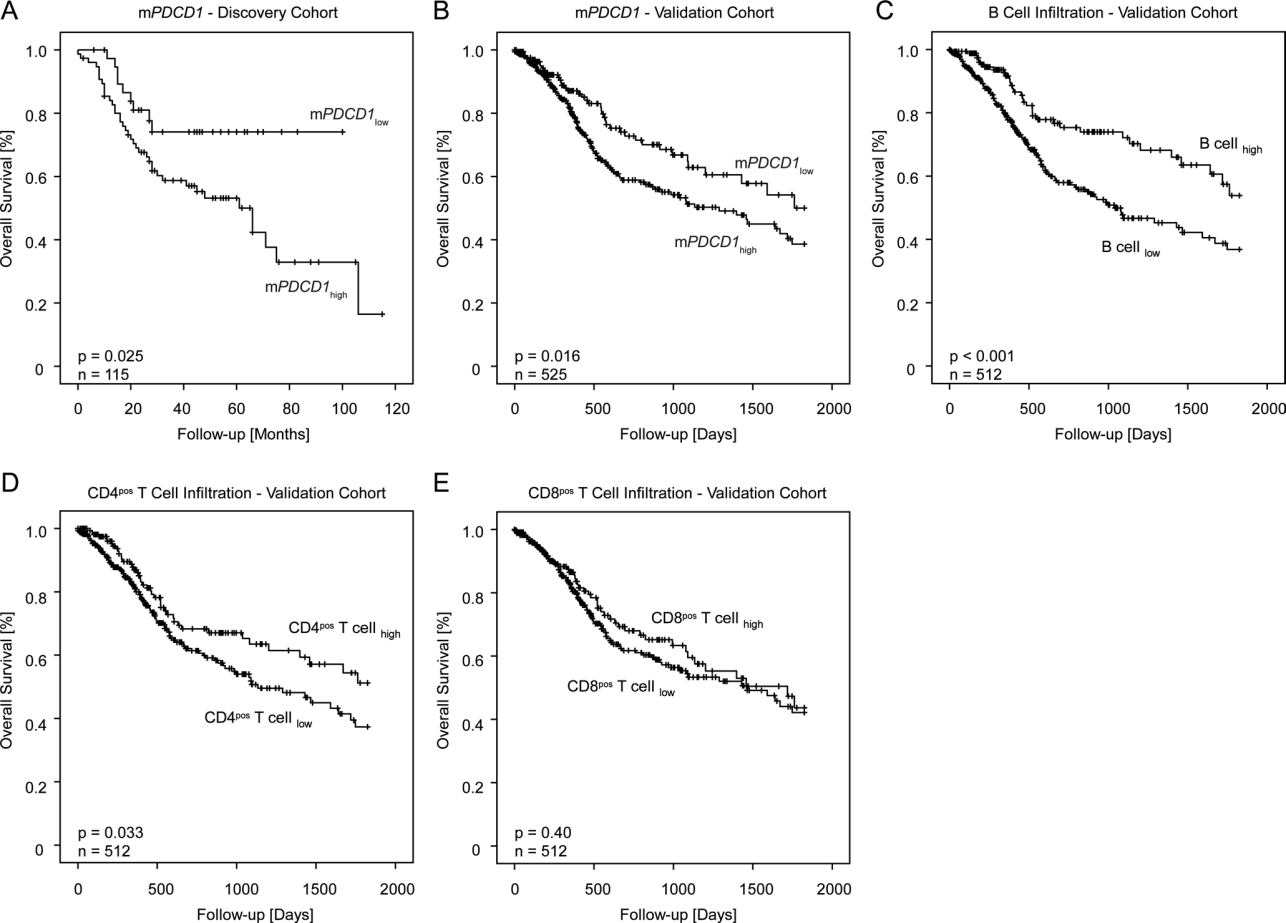
Kaplan-Meier analysis of overall survival in HNSCC patients in the discovery cohort stratified by *PDCD1* methylation status (**A**). Kaplan-Meier analysis of overall survival in HNSCC patients in the validation cohort stratified by *PDCD1* methylation status (**B**) as wells as stratified by B cell infiltration (**C**), CD4pos T cell infiltration (**D**), and CD8pos T cell infiltration (**E**) in the validation cohort. Patient classification into m*PDCD1*_high_ and m*PDCD1*_low_ as well as into cases with low and high immune cell content were based on the lower (m*PDCD1*) and upper tertile (immune cell infiltrates), respectively.

After dichotomization, the frequency of m*PDCD1*_low_ was significantly higher in oropharyngeal SCC compared to all other categories of tumor location (mouth, hypopharynx, and larynx, Table 1A). In the discovery cohort, dichotomized m*PDCD1*_high_ was significantly associated with shorter overall survival in the univariate Cox proportional hazards model (HR = 2.24 [95%CI: 1.08–4.64], *p* = 0.029) and Kaplan-Meier analysis (*p* = 0.025, Figure 1A).

### *PDCD1* promoter methylation in HNSCC patients (validation cohort)

Since PD-1 expression has been mainly observed in immune cells [15], differential m*PDCD1* seems to reflect changes in the lymphocyte compartment. In the validation cohort, m*PDCD1* was related to the content of inflammatory cells in the tumor samples. According to histological data provided by The Cancer Genome Atlas (TCGA) Research Network, tumor samples were on average (mean) composed of 76.3% tumor cells (95%CI: 74.4–78.2%), 3.5% normal cells (95%CI: 2.4–4.5%), and 7.1% tumor infiltrating lymphocytes (95%CI: 6.0–8.3%). Subtypes of tumor infiltrating lymphocytes in the TCGA cohort as assessed by Li *et al*. [20] were correlated with m*PDCD1*. Tumor infiltrating B lymphocytes as well as CD4^pos^ and CD8^pos^ T lymphocytes inversely correlated with m*PDCD1* (r = –0.175; *p* < 0.001 for B lymphocytes, r = –0.118; *p* = 0.007 for CD4^pos^ T lymphocytes, and r = –0.095; *p* = 0.031 for CD8^pos^ T lymphocytes, *n* = 514 for all, Table 2).

The expression of the major PD-1 ligand PD-L1, encoded by *CD274*, is vital for anti-tumor immune tolerance, enabling the tumor cells to escape from T cell attacks. In the series under investigation, promoter methylation of CD274 (m*CD274*) significantly correlated with m*PDCD1* in tumor samples (r = 0.123; *p* = 0.005; *n* = 527). Further, m*CD274* significantly correlated inversely with infiltrating CD8^pos^ T lymphocytes (r = –0.136; *p* = 0.002; *n* = 514).

### Clinicopathological correlation (validation cohort)

The analysis of clinicopathological parameters revealed a significant positive correlation of m*PDCD1* with age at initial diagnosis and the pT category, while a significant negative correlation was seen with tumor grade (Table 2). P16 expression, as a surrogate marker for HPV association of the tumor, combined with HPV *in situ* hybridization data obtained by the TCGA network showed a strong association of m*PDCD1* with HPV status (Table 2). Regarding immune cell infiltration, localization and tumor grade were associated with varying immune cell densities of all components (Table 1B), whereas the pT category was associated with alterations in B and CD4^pos^ T lymphocyte densities. A positive HPV status was associated with increased B and CD8^pos^ T lymphocytic infiltrates.

### *PDCD1* promoter methylation and survival analyses (validation cohort)

Subsequently, we analyzed whether m*PDCD1* and the analysis of immune cells allow for a risk stratification of patients. In the univariate Cox proportional hazard model, both continuous values of immune cell content (HR = 0.06 [95%CI: 0.01–0.38], *p* = 0.003 for B lymphocytes, HR = 0.04 [95%CI: 0.01–0.33], *p* = 0.002 for CD4^pos^ T lymphocytes, and HR = 0.30 [95%CI: 0.09–0.97], *p* = 0.045 for CD8^pos^ T lymphocytes) and m*PDCD1* (HR = 2.52 [95%CI: 1.58–4.04], *p* < 0.001) served as strong prognostic factors (Table 3). Accordingly, patients classified as suffering from hypermethylated (m*PDCD1*_high_) tumors showed a significantly worse overall survival (HR = 1.54 [95%CI: 1.08–2.21], *p* = 0.017) compared to patients with hypomethylated (m*PDCD1*_low_) tumors. The prognostic value of dichotomized m*PDCD1* was further confirmed by Kaplan-Meier analyses (Χ^2^ = 5.76, *p* = 0.016 for m*PDCD1*_low_ and m*PDCD1*_high_, respectively; Figure 1B). For Kaplan-Meier analyses of immune cell infiltrates see Figure 1C–1E).

**Table 3:** Univariate and multivariate Cox proportional hazards analyses on overall survival in HNSCC patients treated by radical surgical resection (validation cohort)

		Univariate Cox proportional hazards		Multivariate Cox proportional hazards*	
Variable	*n*	*p*-value	Hazard ratio [95% CI]	*p*-value	Hazard ratio [95% CI]
pT (pT_3-4_vs. pT_1-2_)	459	< 0.001^¥^	1.95 [1.35–2.82]	0.010	1.91 [1.17–3.13]
pN (pN_2_ *vs*. pN_1_ *vs*. pN_0_)	414	0.001^¥^	1.45 [1.18–1.79]	0.002	1.41 [1.13–1.76]
Tumor grade (WHO 2006)	499	0.82	1.03 [0.81–1.32]	NA	NA
Distant Metastases (clinical staging, cM_1_vs. cM_0_)	521	0.19	1.00 [0.97–1.01]	NA	NA
Surgical margin (R_1_ *vs*. R_0_)	460	0.067	1.48 [0.97–2.26]	NA	NA
HVP status (positive *vs*. negative)	107	0.53	0.66 [0.18–2.45]	NA	NA
History of smoking (smokers *vs*. non-smokers)	508	0.28	1.25 [0.84–1.87]	NA	NA
*PDCD1* methylation (dichotomized, cut-off 23.1%)	521	0.017^¥^	1.54 [1.08–2.21]	NA	NA
*PDCD1* methylation (logarithmic continuous variable)	521	< 0.001^¥^	2.52 [1.58–4.04]	0.011	2.14 [1.19–3.84]
B lymphocytes	514	0.003^¥^	0.06 [0.01–0.38]	0.66	0.48 [0.02–12.11]
CD4^pos^ T lymphocytes	514	0.040^¥^	0.04 [ 0.01–0.33]	0.040	0.04 [0.00–0.86]
CD8^pos^ T lymphocytes	514	0.045^¥^	0.30 [0.09–0.97]	0.28	2.68 [0.45–15.86]

Multivariate Cox proportional hazard analysis was conducted including only variables that showed significance in univariate analysis (pT, pN, *PDCD1* methylation [logarithmic continuous variable], B lymphocytes, CD4^pos^ T lymphocytes, and CD8^pos^ T lymphocytes). Data for multivariate analysis was available for *n* = 402 patients.

^¥^ significant feature; NA: not available.

Since m*PDCD1* did not correlate with major clinicopathological parameters, age and pT category excluded, we hypothesized that it might serve as an independent prognostic factor in HNSCC. In multivariate Cox Proportional Hazards analysis, continuous m*PDCD1* (HR = 2.14 [95%CI: 1.19–3.84], *p* = 0.011) and continuous CD4^pos^ T lymphocytic infiltrates (HR = 0.04 [95%CI: 0.00–0.86], *p* = 0.040) remained independent significant prognostic factors for overall survival when tested together with parameters with significant prognostic value in univariate Cox proportional Hazard analyses (pT,pN categories, B cell, and CD8^pos^ T lymphocytic infiltrates, Table 3).

## DISCUSSION

In the present study, we perceived m*PDCD1* as a surrogate marker for immune cell infiltration. It was shown to have a considerable impact on the course of HNSCC patients. High m*PDCD1* levels, applied as single or combined values, were linked to a significantly shorter overall survival after surgical resection. In univariate and multivariate Cox proportional hazards analysis, both immune cell infiltration and m*PDCD1* methylation as continuous variables further served as highly significant prognostic factors in HNSCC, thereof m*PDCD1* and CD4^pos^ T lymphocytic infiltrates being independent and as powerful as pT and pN categories.

Aberrant promoter methylation of established or candidate tumor suppressor genes, in addition, has been shown to be essential for HPV-induced carcinogenesis in HNSCC [21], indicating the potential value of methylation as prognostic biomarker in this tumor entity [22]. Accordingly, m*PDCD1* was significantly lower in HPV^+^ HNSCC and in tumors occurring in non-smokers, suggesting a major role in the PD-1 driven adaptive immune resistance in the subgroup of HPV^+^ HNSCC. While high B and CD8^pos^ T lymphocytic immune cell infiltrates were associated with HPV persistence, neither CD4^pos^ T lymphocytes nor tumor-associated macrophages and dendritic cell infiltrates were related to HPV status. In this context, it may be of importance that HPV^+^ HNSCCs contain a distinct population of PD-1 high expressing CD8 ^pos^ T cells [14], while the frequency of myeloid derived suppressor cells and tumor activated macrophages seems to be independent of HPV infection status in HNSCC [23]; an observation that could be reproduced in the present dataset.

Epigenetic alterations are involved in the regulation of gene expression in key biological processes, i.e. development, differentiation, alternative splicing, and genetic imprinting of various cell types [24–26]. It seems reasonable, however, that the differential variation of m*PDCD1* was secondary to alterations in the immune cell content in the present study; all the more so since epigenetic *PDCD1* promoter control of PD-1 expression has been described for human T lymphocytes [17, 18]. These data are in line with the observation that m*PDCD1* is roughly 100% in non-immunogenic cancer epithelium like the prostatic adenocarcinoma [19]. Nevertheless, it may be premature to rule out a tumor-intrinsic role of m*PDCD1* in HNSCC. In fact, it must be noted that such associative analysis should be interpreted with caution and used to neither assume nor reject a direct regulation without additional experimental support.

Prolonged viral infections and cancer lead to chronic antigen exposure and can induce high expression levels of PD-1. PD-1 regulates the exhaustion of antigen-specific T cells, and T cells with high PD-1 expression consequently lose the ability to eliminate cancer. (see [15] for rev). In this context, Youngblood *et al*. reported that the *PDCD1* promoter was fully demethylated in antigen-experienced PD-1^high^CD8^pos^ T cells, whereas methylation levels were significantly lower in antigen-experienced PD-1^low^CD8^pos^ T cells [18]. We therefore speculated that the degree of T cell exhaustion may be very well reflected by their methylation levels of the *PDCD1* promoter. Since *PDCD1* promoter methylation was higher in tumors containing minor amounts of infiltrating lymphocytes and cancers with adverse prognosis, our findings are in line with the observation that tumors with dense lymphocytic infiltrates, like HPV-associated HNSCCs [27], altogether have a favourable course of disease. In addition, *CD274* promoter methylation significantly correlated with *PDCD1* methylation, suggesting that epigenetic regulation of the PD-1 receptor may be paralleled by PD-L1 induction in tumor tissue.

Not only is the PD1/PD-L1 axis involved in the reduction of immune effector responses in tumors, but it also affects T cell responses in secondary lymphoid tissues, moving the balance from T cell activation towards antigen tolerance. This modulation of the immune system is mediated via regulatory T cells (TRegs), a subpopulation of T cells which maintain self-tolerance and are also found in the immediate vicinity of tumors. Paradoxically, although inhibiting effector T cells, these receptors seem to enhance TReg cell activity or proliferation [15]. In this context, it would be crucial to define thresholds for *PDCD1* methylation and consecutive PD-1 expression on T cells that establish anti-tumor immunity. With regards to the limitations of the present study, the application of our approach is generally not sufficient to precisely determine the levels of *PDCD1* methylation and PD-1 expression in infiltrating immune cells, i.e. elucidation of *PDCD1* in certain T lymphocytic strains, among others CD8^pos^ and TRegs. To constrain the issue more profoundly, a different strategy needs to be adopted, whereby additional observations are collected by tissue digestion and detailed reworking on the microenvironment of the tumor. However, these studies need to be planned prospectively and are subject of our ongoing studies.

Although further mechanistic studies are clearly warranted in order to fully characterize the role of PD-1 expression in HNSCC, our results might imply that the densities of B and CD4^pos^/CD8^pos^ T lymphocytic infiltrates may be easily estimated measuring m*PDCD1* in HNSCC. This finding may be of significance for the future application of immunotherapies in HNSCC patients. Moreover, m*PDCD1* might potentially serve as a predictive biomarker for the response to immunotherapies targeting the PD-1/PD-L1 axis.

Recent data obtained in the KEYNOTE-012 expansion cohort have shown that, similar to the initial HNSCC cohort of the trial, a higher response to pembrolizumab was observed in patients with HPV^+^ versus HPV^-^ HNSCC [12]. However, emerging evidence supports the use of PD-1–targeted therapies to treat both groups of patients [14, 23]. Besides PD-L1 expression on the surface of tumor cells, mutational load, and the intensity of intratumoral CD8^pos^ T cell infiltrates have each been proposed as distinct biomarkers of response to PD1 targeted therapies [13]. For HNSCC, m*PDCD1* as a surrogate marker for immune cell infiltration may be of special value, since differential PD-L1 expression has failed to discriminate between patients prone to therapy failure and those with a reasonable response to [12, 28]. The activation of the co-inhibitory checkpoint molecule PD-1 in T lymphocytes and expansion of myeloid derived suppressor cells are considered the major mechanisms for tumors to escape from immune surveillance [29]. However, the latter factors are functionally interrelated and may often be found simultaneously in individual tumors. Therapies targeting the PD-1/PD-L1 pathway have shown excellent results in HNSCC. Reliable biomarkers predicting the response to treatment, however, are still lacking. So far, no data are available on the frequency or significance of promoter methylation regarding this immunomodulatory pathway. Our study is the first to show that *PDCD1* methylation predicts the outcome in HNSCC patients, accordingly, potentially aiding the identification of HNSCC patients who might benefit from adjuvant treatment after radical surgical resection, particularly in the context of immunomodulatory therapies. Furthermore, *PDCD1* methylation was shown to be associated with a HPV^+^ status, suggesting a major role of the PD-1 driven adaptive immune resistance in this tumor subgroup.

## MATERIALS AND METHODS

### Patients, ethics and clinical endpoint

### Discovery cohort

Formalin-fixed and paraffin-embedded (FFPE) specimens from 120 patients diagnosed with HNSCC and having undergone surgical resection at the University Hospital Bonn between 1999 and 2013 were retrospectively included in the discovery cohort. Overall survival was considered as the endpoint of the study.

### Validation cohort

The data from the validation cohort are based upon data generated by TCGA Research Network: http://cancergenome.nih.gov/. The TCGA cohort comprised fresh-frozen tissues from 527 patients with histologically confirmed HNSCC from several international centers involved in the TCGA project. From 50 patients matched normal adjacent tissues were available.

### Clinical Endpoint

Overall survival (OS) was considered as the primary endpoint of the study. OS was censored after 5 years (1825 days). Clinical follow-up was available for 527 individuals.

### Ethics

The study was approved by the Institutional Review Board at the University Hospital of Bonn. The TCGA Research Network obtained informed consent (written) from all patients included in the validation cohort. All experiments were performed in accordance with the relevant guidelines and regulations.

### Sample preparation (discovery cohort)

For the analysis of m*PDCD1*, patient samples were processed according to the InnuCONVERT Bisulfite All-In-One Kit (Analytik Jena, Germany) as previously published [30].

### *PDCD1* quantitative methylation real-time PCR (discovery cohort)

m*PDCD1* was determined by means of a methylation-specific real-time PCR assay targeting the *PDCD1* promoter region [19]. The methylation-specific real-time PCR assay was duplexed with a second assay targeting a CpG-free region within the *ACTB* gene locus and allowing for the quantification of the total DNA, irrespective of its methylation [19]. PCR conditions (buffers, temperature cycling program, real-time PCR instrument) were applied as previously described [31]. The following primers and probes were used: *PDCD1* forward primer, 5′-tcgaagcgaggttagaaatcgtt-3′; *PDCD1* reverse primer, 5′-ccttcaaaaccgaaccgaatat-3′; *PDCD1* probe, 5′-6-FAM-ttggcgcggttgtttggtttcgaga-BHQ-1-3′; *ACTB* forward primer, 5′-cccttaaaaattacaaaaaccacaa-3′; *ACTB* reverse primer, 5′-ggaggaggtttagtaagttttttg-3′; *ACTB* probe, 5′-Atto-647N-accaccacccaacacacaataacaaacaca-BHQ-2-3′. Each sample was measured in triplicate with an input of 25 ng of bisulfite-converted FFPE tissue DNA as quantified via UV. As a calibrator 3 ng of bisulfite-converted artificially methylated DNA (CpGenome Universal Methylated DNA, Merck Millipore, Billerica, MA, USA) was used. m*PDCD1* was calculated with the ΔΔCT method as described earlier [31]).

### Data processing (validation cohort)

TCGA methylation data were created by the TCGA Research Network (http://cancergenome.nih.gov/) using the Infinium HumanMethylation450 BeadChip (Illumina, Inc., San Diego, CA, USA). Methylation values for each bead pair comprised of a variant specific for the methylated and the unmethylated status, respectively, and were calculated by the formula 100*bead_intensity_methylated/(bead_intensity_methylated+ bead_intensity_ unmethylated) as previously described [32]. Data of level 2 from the TCGA Head and Neck Squamous Cell Carcinoma (TCGA-HNSCC) cohort were downloaded from the TCGA webpage. The five beads (cg20805133, cg00795812, cg27051683, cg17322655, cg03889044) located within the upstream CpG-island located in the *PDCD1* promoter (Supplementary Figure 1) were selected and the mean methylation value of all five bead pairs from one patient sample was computed. For the quantification of *CD274* promoter methylation, bead cg19724470 was chosen [33]. Data on immune cell infiltrates were adopted from [20].

### Dichotomization of continuous methylation values and immune-cell infiltration

For detailed clinicopathological correlation and survival analyses, m*PDCD1* values as well as quantitative data on immune cell infiltrates were considered as continuous variables and as dichotomized variables to obtain qualitative results. For the dichotomization of DNA methylation values, patients were stratified according to m*PDCD1* tertiles (T_1–3_), in analogy to other three-level graduation systems commonly used in pathologic classifications (e.g. immunohistochemical staining intensity in immunoreactive scores). The cut-off was set between T_1_ (m*PDCD1*_low_) and T_2/3_ (m*PDCD1*_high_) and was 21.4% in the discovery cohort and 23.06% in the validation cohort, respectively. For immune cell infiltrates (B lymphocytes and T lymphocytes), the cut-off was set in an analogous manner between between T_1/2_ (B cell_low_, CD4^pos^_low_, CD8^pos^_low_) and T_3_ (B cell_high_, CD4^pos^_high_, CD8^pos^_high_).

### Statistical analyses

Statistical analyses were performed using SPSS, version 22.0 (SPSS Inc., Chicago, IL). Statements regarding potential correlations of specific histological findings were made using the Spearman correlation coefficient. Comparisons were performed using the Wilcoxon-Mann-Whitney *U* test, the Kruskal-Wallis test, and the χ^2^ – test/Fisher Exact test. Survival analyses were performed using the Kaplan-Meier method, and differences between the patient groups were testes by the log rank test. Hazard ratios (HR) were calculated using univariate and multivariate Cox proportional hazards models. Continuous m*PDCD1* values were logarithmized to base 2. *P*-values less than 0.05 were considered statistically significant.

## SUPPLEMENTARY MATERIALS FIGURES AND TABLES


