# Supplementary material for: PDCD1 (PD-1) promoter methylation predicts outcome in head and neck squamous cell carcinoma patients
Source: Oncotarget. 2017 Apr 21;8(25):41011–20. doi: 10.18632/oncotarget.17354 (PMC5522222; doi:10.18632/oncotarget.17354)
Supplement: Supplementary file 1 [file oncotarget-08-41011-s001.pdf]

PDCD1 (*PD-1*) promoter methylation predicts outcome in head and neck squamous cell carcinoma patients

Supplementary Materials

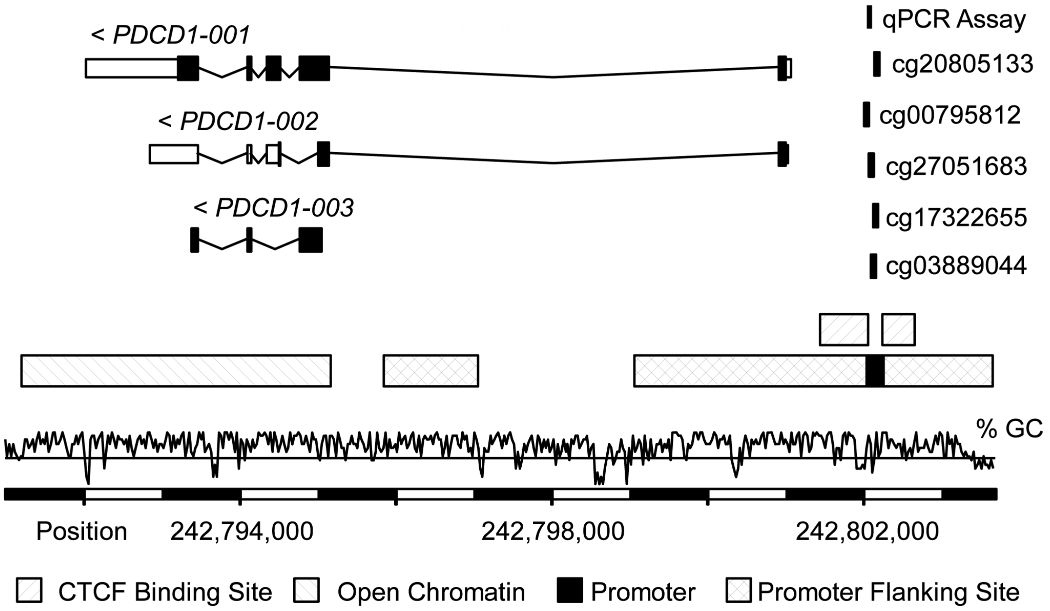

Supplementary Figure 1: Genomic organization of the *PDCD1* gene and location of the analyzed bead pairs from the Infinium HumanMethylation450 BeadChip. Gene organization is based on the Genome Reference Consortium Human Build 37 patch release 13 which is illustrated using the Ensemble genome browser (http://www.ensembl.org). The analyzed methylation specific bead pairs as well as the methylation-specific qPCR assay located in the promoter region are annotated. Percentage of GC content, regulatory elements (CTCF binding site and promoter) and different mRNA variants are shown.
